# DESIGNING AND DISSEMINATING AN EDUCATIONAL PROGRAM IN OVER-THE-COUNTER HEARING TECHNOLOGY

**DOI:** 10.1093/geroni/igae098.0175

**Published:** 2024-12-31

**Authors:** Yi Shen, Dana Urbanski, Susan Anderson, Adrian Lee

**Affiliations:** University of Washington, Seattle, Washington, United States; University of Tennessee Health Science Center, Knoxville, Tennessee, United States; University of Washington, Seattle, Washington, United States; University of Washington, Seattle, Washington, United States

## Abstract

Over-the-Counter (OTC) hearing aids have been available to the US market since October 2022. These “do-it-yourself” hearing devices were introduced to promote accessibility and affordability of hearing healthcare to primarily older adults with perceived mild-to-moderate hearing loss. To date, many new delivery models of hearing healthcare have emerged in response to the availability of OTC hearing aids. However, without the assistance of a licensed hearing care professional, it is unclear whether older adults who purchase OTC hearing aids will receive sufficient support and assistance to achieve successful OTC hearing aid use. In this presentation, we review recent research to identify areas of unmet need in currently available delivery models for OTC hearing aids. The available evidence suggests the need for and potential benefits of an educational program on OTC hearing technology independent from any device manufacturer that could be delivered to professionals who are not hearing healthcare professionals but who may interact with older adult OTC hearing aid candidates (e.g., community health workers, long-term care staff, pharmacists, and other allied health professionals). To address this need, we conducted an instructional design process with a team of healthcare educators, researchers, audiologists, and instructional designers to create the blueprint of an educational program on OTC hearing technology. Here, we describe our design process for transforming the identified unmet needs into (1) the target audience; (2) the appropriate learning modes for the intended audience; and (3) a list of learning objectives. Additionally, we highlight our plans for implementation and dissemination of the program.

